# Cell Penetrating Capacity and Internalization Mechanisms Used by the Synthetic Peptide CIGB-552 and Its Relationship with Tumor Cell Line Sensitivity

**DOI:** 10.3390/molecules23040801

**Published:** 2018-03-30

**Authors:** Soledad Astrada, Julio Raúl Fernández Massó, Maribel G. Vallespí, Mariela Bollati-Fogolín

**Affiliations:** 1 Cell Biology Unit, Institut Pasteur de Montevideo, Mataojo 2020, 11400 Montevideo, Uruguay; sastrada@pasteur.edu.uy; 2 Department of Genomic, Center for Genetic Engineering and Biotechnology, Cubanacan, P.O. Box 6162, Havana 10600, Cuba; julio.fernandez@cigb.edu.cu; 3 Pharmaceutical Department, Center for Genetic Engineering and Biotechnology, Cubanacan, P.O. Box 6162, Havana 10600, Cuba; maribel.guerra@cigb.edu.cu

**Keywords:** cell penetrating peptide, endocytosis, transduction

## Abstract

CIGB-552 is a twenty-amino-acid novel synthetic peptide that has proven to be effective in reducing tumor size and increasing lifespan in tumor-bearing mice. Such capability is conferred by its cell-penetrating peptide character, which allows it to enter cells and elicit a pro-apoptotic effect through its major mediator, COMMD1 protein. Cell-penetrating peptides are able to use different internalization mechanisms, such as endocytosis or direct transduction through the plasma membrane. Although CIGB-552 cytotoxicity has been evaluated in several non-tumor- and tumor-derived cell lines, no data regarding the relationship between cell line sensitivity, cell penetrating capacity, the internalization mechanisms involved, COMMD1 expression levels, or its subcellular localization has yet been produced. Here, we present the results obtained from a comparative analysis of CIGB-552 sensitivity, internalization capacity and the mechanisms involved in three human tumor-derived cell lines from different origins: mammary gland, colon and lung (MCF-7, HT-29 and H460, respectively). Furthermore, cell surface markers relevant for internalization processes such as phosphatidylserine, as well as CIGB-552 target COMMD1 expression/localization, were also evaluated. We found that both endocytosis and transduction are involved in CIGB-552 internalization in the three cell lines evaluated. However, CIGB-552 incorporation efficiency and contribution of each mechanism is cell-line dependent. Finally, sensitivity was directly correlated with high internalization capacity in those cell lines where endocytosis had a major contribution on CIGB-552 internalization.

## 1. Introduction

Cell-penetrating peptides (CPPs) are defined as small molecules that are mainly cationic, ranging from 5 to 30 amino acids, capable of penetrating cells with or without receptor mediation [1,2,3]. Although they have been characterized as transport molecules, they are also capable of eliciting effects by themselves. In particular, antimicrobial peptides (AMPs) have already been reported to promote anti-tumor effects, given their capacity to identify malignant cells. Such recognition is based on electrostatic interactions favored by the increase of negatively charged components in the outer surface of malignant cells [4]. CIGB-552 is an example of an AMP-derived synthetic peptide with cell penetrating capacity and the ability to produce cytotoxic effect by increasing apoptosis levels of malignant cells [5,6,7]. CIGB-552 specifically promotes the stabilization of the COMMD1 protein, a small cytosolic protein involved in copper homeostasis, sodium transport, and the nuclear factor kappa-light-chain-enhancer of activated B cells (NF-κB) signaling pathway [5,8]. It has been reported that the CIGB-552 cytotoxic effect is dependent on COMMD1 expression [5]. Internalization mechanisms used by CPPs have been widely studied for several different peptides in a variety of cell lines. However, no data regarding cell penetrating capacity and the mechanisms involved in different cell lines have been produced for CIGB-552. Two different mechanisms have been implied for CPP internalization: endocytosis and direct transduction through the membrane. While endocytosis occurs at 37 °C and is an energy-dependent process, transduction through the plasma membrane can proceed at lower temperatures (4 °C) and is an energy-independent process [1,3,9]. On the other hand, CIGB-552 cytotoxicity has been characterized in several cell lines, including non-tumor-derived cell lines, whose sensitivity to the peptide treatment proved to be low, as reflected by the high half maximal inhibitory concentration (IC_50_) values obtained [6,7]. Regarding tumor-derived cell lines, CIGB-552 showed lower IC_50_ values, thus indicating higher sensitivity to peptide treatment [5,6,7]. However, differences were also observed between tumor-derived cell lines considering the variability of tissue origin. Specifically, H460 is considered to be one of the most sensitive cell lines, while MCF-7 is considered to be one of the most resistant so far explored (Table 1) [5,10].

Therefore, the aim of the present work was to evaluate the relationship between the cell penetrating capacity of CIGB-552, the internalization mechanisms involved, and COMMD1 expression on three different tumor-derived cell lines with dissimilar sensitivity to the peptide: MCF-7, HT-29 and H460. As already stated, the IC_50_ for each cell line has been reported, showing that H460 is the most sensitive cell line, MCF-7 is the most resistant cell line, and HT-29 shows an intermediate sensitivity [7]. Our results showed that cell penetrating capacity varies among the three cell lines studied. H460 displayed the highest internalization levels, while MCF-7 presented the lowest internalization capacity. Moreover, CIGB-552 used both endocytosis and transduction as internalization mechanisms, although the contributions of each mechanism varied among the studied cell lines. Finally, we evaluated in situ COMMD1 expression levels, and we found that both MCF-7 and H460 have the highest expression levels. Altogether, our results suggest that H460 sensitivity could be explained by its high internalization capacity of CIGB-552, by using the endocytosis as a preferred mechanism, which in turn favors the interaction between the peptide and its target protein COMMD1 in the endosomal compartment. 

## 2. Results

### 2.1. Cell Penetrating Capacity of CIGB-552 and Internalization Mechanisms Involved

**Figure 1:**
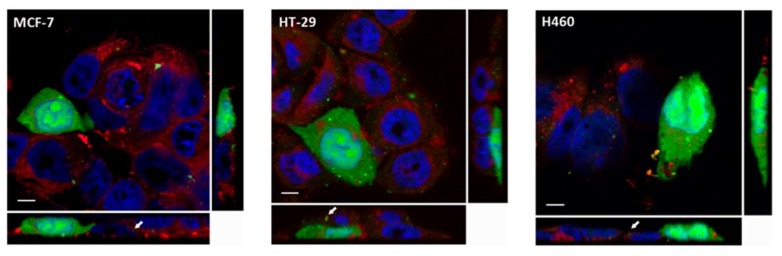
CIGB-552 (50 μM, in green) is capable of penetrating the three cell lines, displaying two different internalization patterns: fully loaded cells and punctuated pattern (arrow) (scale bar = 5 μm). Nuclei were labelled using TOPRO-3 probe (blue) and cell membranes were labelled with CellMask (red).

**Figure 2:**
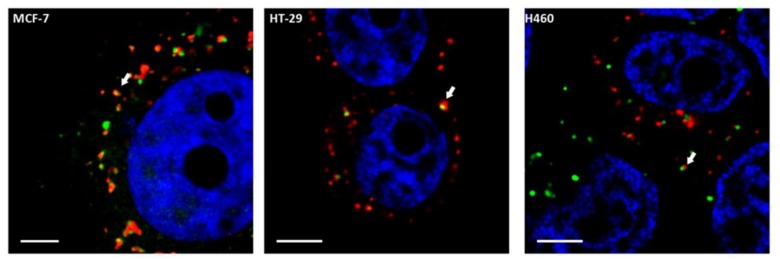
The punctuated pattern observed in CIGB-552 (green) internalization showed partial co-localization with Rab5A-RFP in red (arrow), which is an early endosomal marker, indicating that endocytosis might be one of the mechanism involved in CIGB-552 internalization (scale bar = 5 μm). Nuclei were labelled using TOPRO-3 probe (blue).

Different internalization mechanisms have been described for CPPs, such as endocytosis and direct transduction through the plasma membrane. Both mechanisms differ not only in their energetic requirements, but also in terms of the cellular internalization patterns observed [1,3,9,11,12,13]. While endocytosis displays a punctuated pattern in the cytoplasm as a result of internalization in endocytic vesicles, transduction is characterized by a homogenous distribution of the cytoplasms (fully loaded cells) reaching the nucleus [12]. Therefore, internalization patterns in the three cell lines (MCF-7, HT-29 and H460) were evaluated using confocal microscopy and fluorescein isothiocyanate (FITC)-conjugated CIGB-552. We observed that CIGB-552 is capable of penetrating the three cell lines tested showing two different internalization patterns, fully loaded cells (FLC) and a punctuated pattern (Figure 1), demonstrating the occurrence of different internalization mechanisms. Moreover, an early endosome marker, Rab5A, a small GTPase [14,15], was used to define whether the punctuated pattern observed correlated with endocytic vesicle internalization. We found partial co-localization with Rab5A (Figure 2), indicating that endocytosis might be one of the mechanism involved in CIGB-552 internalization.

**Figure 3:**
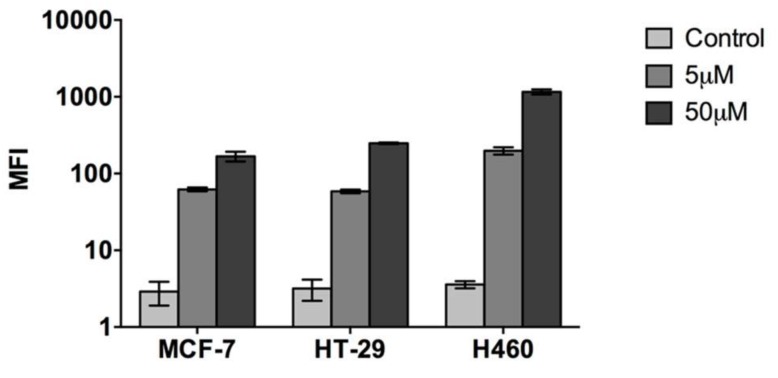
CIGB-552 cell penetrating capacity was evaluated by flow cytometry in the three cell lines used. Median fluorescence intensity values (MFI) showed that H460 cell line possess the highest internalization levels at both concentrations whereas HT-29 and MCF-7 showed similar levels of internalization. Results showed a direct correlation between concentration increase and internalization levels.

**Figure 4:**
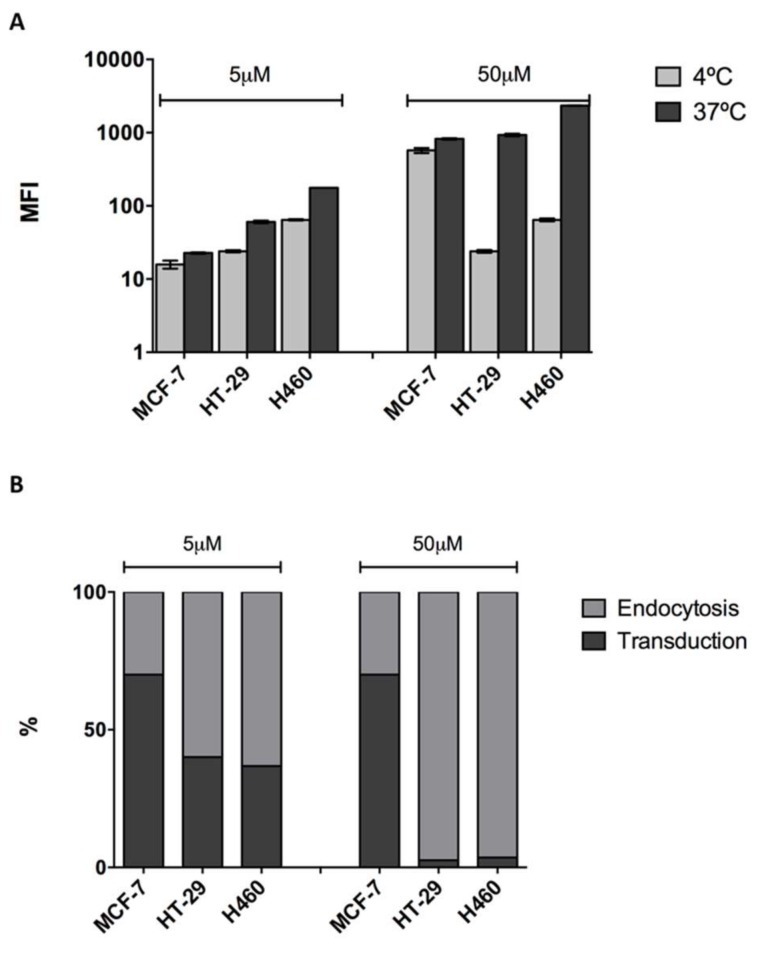
Temperature effect on CIGB-552 internalization evaluated by flow cytometry. (**A**) A reduction in the internalization levels of CIGB-552 was observed at both concentrations used in all the cell lines when assays were performed at 4 °C. However, reduction levels were not the same between cell lines. Both HT-29 and H460 displayed a higher reduction in internalization levels as reflected by values obtained at 37 °C and 4 °C; (**B**) Estimated contribution of each internalization mechanism as a percentage (%) varied among cell lines. The contribution of transduction in MCF7 cell line is distinctively higher than that occurring in the HT-29 and H460 cells.

In order to quantitatively assess the cell penetrating capacity of CIGB-552, we used flow cytometry. Cells were incubated with two different peptide concentrations, 5 and 50 μM, for 1 h at 37 °C, and then median fluorescent intensity (MFI) values were evaluated. Although clear differences were observed in all cell lines between treated (CIGB-552) and non-treated cells (control), cell penetrating capacity did differ among them. The H460 cell line had the highest internalization levels both at 5 μM and 50 μM concentrations, whereas HT-29 and MCF-7 showed similar levels of internalization (Figure 3). Results showed a direct correlation between concentration increase and internalization levels. Next, we decided to evaluate internalization mechanisms using a more functional approach. As already stated, the conditions needed for endocytosis and transduction to occur are different. While endocytosis is an energy-dependent process that proceeds only at 37 °C, transduction is an energy-independent process that can take place even at lower temperatures, such as 4 °C [1,3]. Therefore, by performing internalization assays at both temperatures, 37 °C and 4 °C, we evaluated the contribution of endocytosis and transduction in each cell line. We must point out that given the fact that two different mechanisms appear to be responsible for the internalization of CIGB-552, the use of endocytosis inhibitors was dismissed. Although Genistein and Chlorpromazine are widely used as endocytosis inhibitors, they have proven to alter membrane properties, thus interfering with transduction mechanisms [16,17,18]. A reduction in the internalization of CIGB-552 at 4 °C was observed in each cell line, regardless of the concentration used (Figure 4A). However, the effect of temperature on the internalization was not the same among the cell lines. HT-29 and H460 displayed a higher reduction in internalization levels compared to those observed for the MCF-7 cell line (Figure 4A). Considering that endocytosis and transduction may occur simultaneously at 37 °C, we assumed that the values obtained at this condition represent the total amount of CIGB-552 internalization. Therefore, we estimated the contribution of each mechanism as a percentage by referring values obtained at 4 °C (transduction) to those observed at 37 °C (transduction plus endocytosis). In the MCF-7 cell line, the contribution of the transduction mechanism is clearly higher, at more than 50%; whereas for HT-29 and H460, the contribution of the transduction mechanism represents less than 50% (Figure 4B). Notably, for HT-29 and H460 cells there is a remarked reduction in transduction processes at 50 μM. Meanwhile, transduction levels in MCF-7 appear to be quite stable, independently of the peptide concentration used (Figure 4B).

### 2.2. Evaluation of Phosphatidylserine as a Transduction Marker

**Figure 5:**
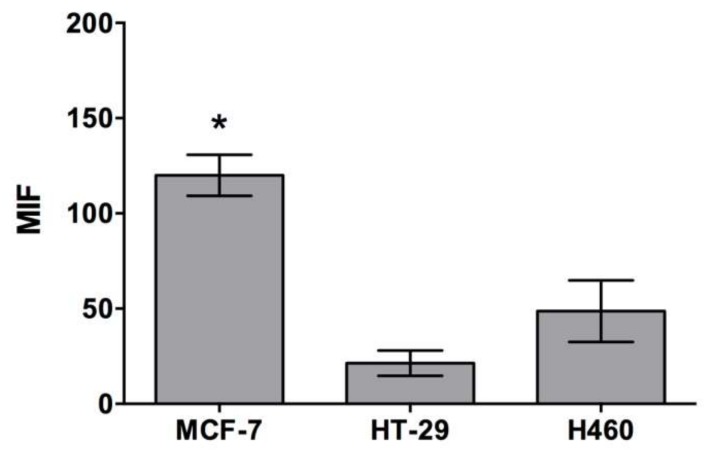
Phosphatidylserine (PS) may play a role in the internalization mechanism of CIGB-552 favoring transduction. To evaluate PS levels, Annexin V was used as a marker and further evaluated its levels by flow cytometry. Values obtained showed that MCF-7 possess the highest expression of PS (* Mann-Whitney U Test, *p* < 0.05).

Electrostatic interactions play a key role in CPPs internalization mechanisms, since the negatively charged components of the membrane interact with the positive residues contained in the peptide sequence. Such interactions enhance the accumulation of peptides on the outer cell surface, which are necessary for the transduction mechanism to occur [11]. Anionic lipids such as phosphatidylserine (PS), whose levels are altered in tumor-derived cell lines [19,20], may confer a negative character to the membrane. Therefore, the level of PS on each cell line surface was evaluated by using FITC-conjugated Annexin V and flow cytometry. MCF-7 proved to be the cell line with the highest surface expression of PS compared to those observed in H460 and HT-29 cell lines (Figure 5). This result reinforces the finding that the transduction mechanism is favored in MCF-7.

### 2.3. COMMD1 Localization and Expression

**Figure 6:**
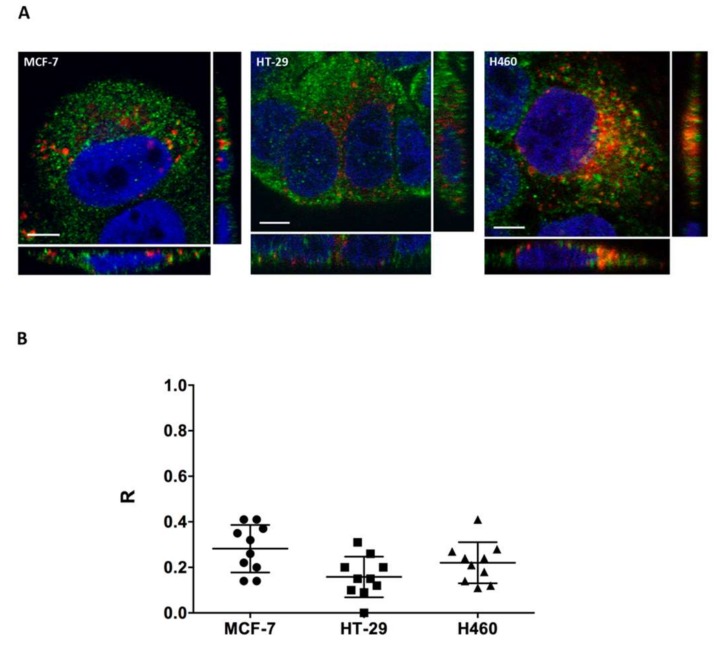
(**A**) COMMD1 is partially located at the endosomes based on the co-localization of COMMD1 (green) and Rab5A (red) observed in the three cell lines used (scale bar = 5 μm); (**B**) co-localization between COMMD1 and Rab5A was evaluated by image analysis. All three cell lines analyzed showed similar levels of co-localization between Rab5A and COMMD1, as expressed by Pearson’s coefficient (R). COMMD1 in green, Rab5A in red and nuclei in blue.

**Figure 7:**
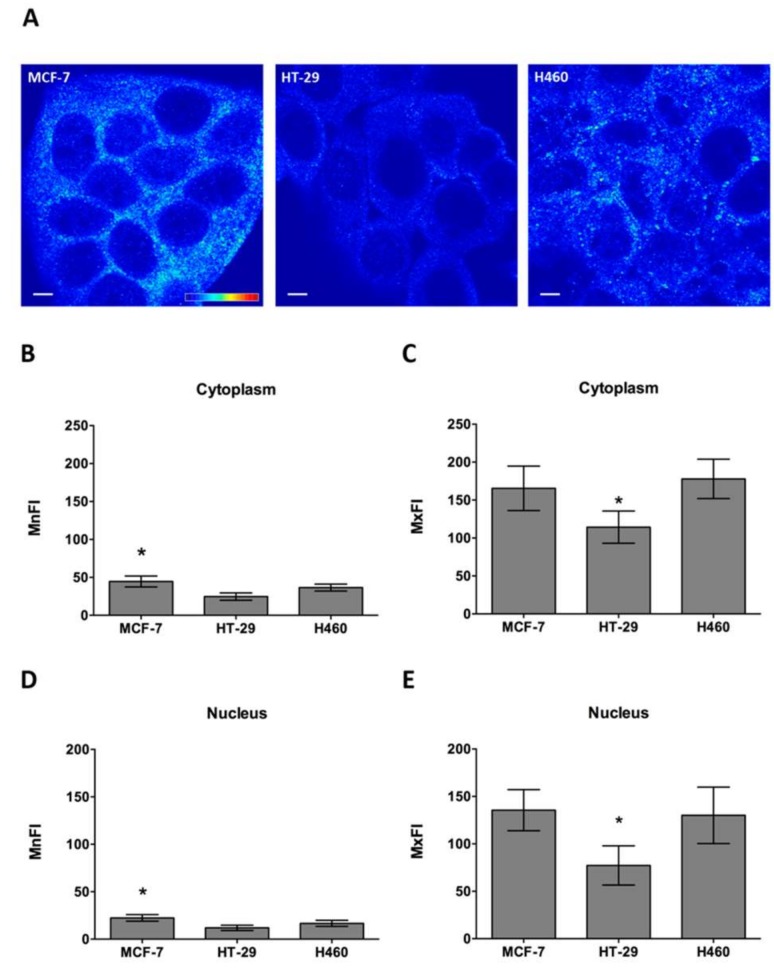
COMMD1 in situ protein levels were evaluated by immunodetection. (**A**) Differences in COMMD1 levels were observed between cell lines using pseudocolor imaging; (**B**,**D**) Mean fluorescence intensity (MnFI) was measured in both nuclei and cytoplasm of 10 single confocal planes for each cell lines. Results obtained showed that MCF-7 appeared to be the cell line with highest amount of COMMD1, followed by H460, whereas HT-29 displayed the lowest levels of COMMD1 in situ; (**C**,**E**) Considering the maximum fluorescence intensity values (MxFI), a similar pattern was observed, in which MCF-7 and H460 had the highest amount of COMMD1, both at the cytoplasm and nuclei (scale bar = 10 μm). * Mann-Whitney U Test, *p* < 0.05.

Cell line sensitivity to the CIGB-552 peptide does not only depend on cell line penetrating capacity of CIGB-552, but also on the presence of COMMD1. It has already been reported that CIGB-552 cytotoxic effect depends on COMMD1 expression, which induces apoptosis [5]. Having proved that endocytosis is one of the internalization mechanisms used by CIGB-552, we wanted to explore whether localization of COMMD1 at endosomal compartments was similar in the three cell lines used, thus favoring the interaction between the peptide and its target protein [21]. We found that COMMD1 was partially located at the endosomes in all three cell lines, as demonstrated by COMMD1 and Rab5A co-localization (Figure 6A). Image analysis showed similar levels of co-localization between Rab5A and COMMD1, as expressed by Pearson’s coefficient (R) (Figure 6B). Therefore, no bias on COMMD1 endosomal localization was observed between cell lines, which may account for differences in sensitivity. However, COMMD1 in situ protein expression levels may indeed explain sensitivity differences observed between cell lines. By using COMMD1 in situ immunodetection, we analyzed the expression levels in cell lines both in the cytoplasm and nucleus. COMMD1 expression levels observed in confocal images varied between cell lines (Figure 7A). Quantitative analysis of COMMD1 expression at the cytoplasm showed that mean fluorescence intensity (MnFI), as well as maximum fluorescence intensity (MxFI), were higher in MCF-7, followed by the H460 cell line, while HT-29 displayed the lowest intensity values (Figure 7B,C). Similar results were obtained at the nucleus, where MCF-7 and H460 showed the highest intensity levels (Figure 7D,E). Overall these results indicate that expression of COMMD1 is higher in MCF-7 and H460.

### 2.4. In Situ Interaction between COMMD1 and CIGB-552

**Figure 8:**
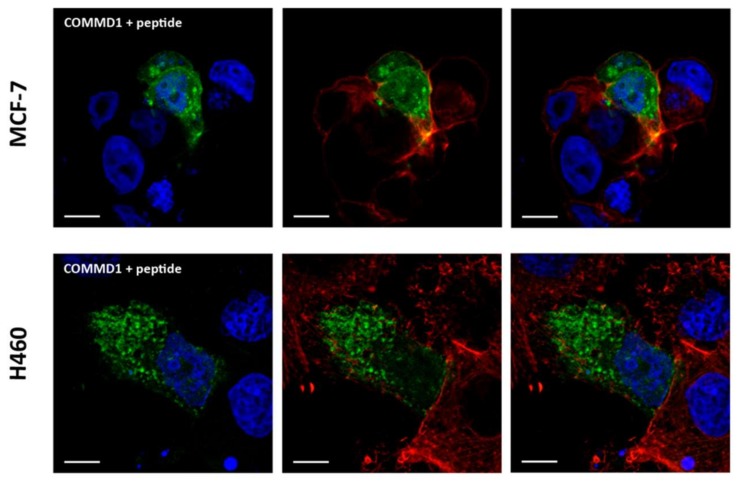
Protein complementation assays were performed between the peptide and COMMD1 protein. MCF-7 and H460 cell lines were co-transfected and 24 h later, in situ interaction was evaluated by confocal microcopy. Both cell lines show the interaction between the peptide and its target protein, resulting in Venus protein expression (green). Nuclei were labelled using TOPRO-3 probe (blue) and actin cytoskeleton was labelled with phalloidin (red) (scale bar = 10 μm).

Interaction between COMMD1 and CIGB-552 has been previously reported by pull down and competitive enzyme-linked immunosorbent assay [5,10]. However, such an interaction has never been demonstrated in a physiological environment such as within cells. Therefore, we selected a protein complementation strategy in which two plasmids containing both the peptide and COMMD1 protein fused to a portion of a reporter protein (Venus, a green fluorescent protein variant). If interaction between the two components actually occurs, the reporter protein is reconstituted, and fluorescence emission is detected. Since CIGB-552 is a synthetic peptide that possess modified amino acids (D amino acids), which cannot be translated inside cells, we decided to use L2 peptide instead. L2, represents the primary sequence that has been modified in order to generate a more stable peptide, CIGB-552 peptide [5,6] (Table 2). Interaction between L2 and COMMD1 protein has also been previously confirmed by pull down assay [5]. In both cell lines, fluorescence emission of Venus protein was observed indicating that the L2 peptide and COMMD1 protein are likely to interact in both the MCF-7 and H460 cell lines (Figure 8).

## 3. Discussion

Despite the efforts that have been made over recent decades, cancer still constitutes one of the main causes of death worldwide. Peptides as therapeutic agents have arisen as an attractive strategy, given the advances provided in synthesis, lack of toxic derivatives and their cell penetrating capacity [22]. CIGB-552 is an example of an AMP-derived synthetic peptide capable of eliciting a cytotoxic effect on malignant cells [5,6,7]. Its action is mediated by the COMMD1 protein, the expression of which is stabilized by CIGB-552 and negatively regulates the NF-κB pathway, thus promoting apoptosis [5]. Data regarding CIGB-552 cytotoxic potential on several cell lines has been documented. However, no data regarding a comparative analysis between cell line sensitivity and CIGB-552 cell penetrating capacity has previously been reported. Therefore, we decided to characterize internalization capacity of different cell lines and the mechanisms involved in order to explain their sensitivity to CIGB-552 treatment. Understanding such properties in cell lines with diverse tissue background may help to predict the effectiveness of CIGB-552 treatment in different tumor types. As we have already stated, two internalization mechanisms have been described for CPPs: endocytosis and transduction [1,3,23]. Both differ not only in their energetic and temperature requirements but also in their cellular internalization patterns. Endocytosis displays a punctuated pattern, while transduction displays a homogeneous distribution throughout the cytoplasm [9,12]. Such patterns have already been effectively correlated with internalization mechanisms in CIGB-552. We have previously reported the occurrence of endocytosis and transduction as internalization mechanisms for CIGB-552 in the MCF-7 line, where the two patterns were also identified [10]. Our results show that both patterns are also present in HT-29 and H460, as previously observed for the MCF-7 cell line, though no differences for FLC frequency were found between the cell lines. On the other hand, endocytosis evidence was provided in the three cell lines by partial co-localization of CIGB-552 with an early endosome marker, Rab5A. Cell penetrating capacity and the contribution of each mechanism to peptide internalization will depend not only on the physicochemical properties conferred by the amino acid sequence present in the peptide, but also in its capacity to interact with the cell membrane components [23,24]. In order to assess such capacity and the occurrence of each mechanism in the internalization of CIGB-552, we selected a more quantitative and functional approach evaluating the incorporation of FITC-conjugated CIGB-552 by flow cytometry. In all cases, incubation with Trypan Blue was performed previous to data acquisition, thus making it possible to measure the peptide that was actually incorporated inside the cells [25]. It has been reported that differences in the internalization mechanisms used could be determined by the abundance of peptide in the outer surface of the cell. At low concentrations, endocytosis is promoted, whereas high concentrations are favorable for the occurrence of transduction, since a threshold concentration is needed to promote the transient destabilization of the cell membrane [9,13,23,26]. Therefore, two different concentrations (5 μM and 50 μM) were selected to evaluate CIGB-552 incorporation. The H460 cell line showed the highest incorporation levels among the three cell lines, with the MCF-7 cell line having the lowest levels of CIGB-552 incorporation at both concentrations tested. Moreover, the internalization increase observed according to the concentration used displayed a linear behavior in the three cell lines. In order to assess the occurrence of both endocytosis and transduction as internalization mechanisms, we decided to perform incorporation assays at different temperatures. As already stated, we discarded the use of endocytosis inhibitors since the membrane perturbation produced by such molecules could increase internalization through transduction as a side effect [16,17,18]. Although internalization was reduced in all cell lines as a consequence of temperature reduction, it was not abolished at any concentration used. H460 and HT-29 cell lines showed the highest sensitivity to temperature reduction, as observed by the decrease in CIGB-552 internalization. We estimated the contribution of each internalization mechanisms and found that CIGB-552 is more prone to using endocytosis as an internalization mechanism in both the HT-29 and H460 cell lines, whereas transduction appears to be the preferred internalization mechanism in MCF-7. Therefore, we proved that not only the internalization capacity but also the internalization mechanism selected is cell line-dependent. As mentioned, properties exhibited by the cell membrane may have an impact on the capacity and internalization mechanism selected. Internalization capacity of CPPs is highly based on their positively charged character which allows them to interact with the negatively charged components available at the outer surface of the cell membrane [11]. Transduction is particularly dependent on electrostatic interaction, since peptide accumulation on the outer surface is needed to trigger a transient membrane destabilization which allows the peptide to enter [26]. Anionic lipids constitute an example of molecules capable of conferring a negative character to the membrane surface. Specifically, PS, which is mainly located in the inner membrane leaflet in normal cells, shows an altered distribution in tumor-derived cell lines [19,20]. Therefore, we decided to explore whether this molecule could account for the internalization mechanisms preference. Accordingly, we observed that MCF-7, for which transduction constitutes the main internalization mechanism, displayed higher levels of PS in its outer surface. However, a more detailed study of the membrane properties is needed to confirm such differences. It has been recently reported that variation in lipid oxidation levels may interfere with CPPs internalization. High oxidation levels increase negative charges of membrane outer surface, thus facilitating CPP penetrability [27]. Moreover, proteoglycans exposed on the outer surface may also constitute a key factor in internalization mechanism selection of CIGB-552. For instance, heparan sulfate has been described as an endocytosis receptor capable of increased CPP internalization [28]. Therefore, heparan sulfate content analysis among different cell lines could provide insights into mechanism selection. Finally, once inside cells, CIGB-552 interacts with COMMD1 in order to elicit its pro-apoptotic effects. Thus, by exploring in situ COMMD1 localization and expression levels in the three cell lines, we aimed to understand their susceptibility to CIGB-552 effects. Although COMMD1 is a widely expressed cytosolic, protein recruitment to the endosomal compartments has also been reported [21]. Considering that the internalization of CIGB-552 takes place partly through the endocytic pathway, localization of both the peptide and its target protein may improve CIGB-552 effects, thus accounting for cell line sensitivity. However, no differences were observed in COMMD1 and Rab5A co-localization levels among the cell lines. On the other hand, differences were observed in COMMD1 protein expression levels. MCF-7 and H460 showed higher and similar expression levels in both cytoplasm and nucleus, while HT-29 had the lowest expression levels as evaluated by fluorescence intensity of confocal microscopy images. Thus, the higher resistance of the HT-29 cell line compared to the H460 cell line is not only due to reduced internalization levels, but is also a consequence of lower COMMD1 expression. On the other hand, the differences observed between MCF-7 and H460 appear to be due to internalization efficacy. However, similar levels of COMMD1 do not account for the occurrence of peptide-protein interaction. In order to evaluate the probability of such interaction taking place in both cell lines, we performed a protein complementation assay. We demonstrated for the first time that in situ interaction between CIGB-552 and COMMD1 protein takes place equally within both cell lines, H460 and MCF-7. Therefore, our results indicate that despite COMMD1 expression levels and its interaction with CIGB-552, the internalization efficacy is determinant in cell sensitivity towards CIGB-552. We have proved that both transduction and endocytosis mechanisms are involved in CIGB-552 internalization. However, the efficacy of both mechanisms is different, leading to different sensitivity to peptide pro-apoptotic effects. The high resistance observed in MCF-7 cell line is a consequence of lower peptide internalization through the transduction mechanism. On the other hand, endocytosis constitutes the most effective way of transporting the CIGB-552 peptide inside cells, thus promoting higher sensitivity towards peptide cytotoxic effects. Altogether, our results show that high sensitivity towards CIGB-552 results from interplay between the internalization mechanism involved, which determines the efficacy of CIGB-552 entrance, and to a lesser extent on COMMD1 abundance.

## 4. Materials and Methods

### 4.1. Reagents and Chemicals

Unless otherwise indicated, all chemicals used were of the highest grade available and were purchased from Sigma-Aldrich (St. Louis, MO, USA). Culture media, fetal bovine serum (FBS), and consumables for cell culture were obtained from Life Technologies (Carlsbad, CA, USA), GE Healthcare, and Greiner. All reagents for peptide synthesis were of synthesis grade. Reagents for chromatography were of high-performance liquid chromatography (HPLC) grade. 

### 4.2. Peptide Synthesis

Peptide CIGB-552 and its analogous metabolites were synthesized on a solid phase and purified by reverse-phase-HPLC to >95% purity on an acetonitrile/H_2_O trifuoracetic acid gradient and confirmed by ion-spray mass spectrometry (Micromass, Manchester, UK) [6]. Lyophilized peptides were reconstituted in apirogenic water for in vitro experiments. The carboxyfluorescein fluorophore was attached selectively by an amide bond to the N-terminus of the peptide sequences during the synthesis of the peptide in solid phase performed using the Fmoc/t-Bu chemistry. The linking is direct to the N-terminus of the peptide; there are no additional residues.

### 4.3. Cell Line and Culture Medium 

MCF-7 (ATCC, HTB-22), HT-29 (ATCC, HT-38) and H460 (ATCC, HTB-177) cells were cultured either in RPMI 1640 or DMEM supplemented with Glutamax and 10% (*v*/*v*) FBS. Cells were routinely propagated in 25 or 75 cm^2^ tissue culture flasks at 37 °C, 5% CO_2_ in a humidified incubator until reaching approximately 70% confluence. Cells were subsequently trypsinized, concentration was adjusted, and used for different experimental settings. Cells were cultured for no longer than 10–15 passages.

### 4.4. Internalization Assay

Cells were seeded in 12-well plates (1 × 10^5^ cells/well) and cultured in either D-MEM or RPMI supplemented with Glutamax, 10% (*v*/*v*) FBS, at 37 °C for 24 h. For those samples where internalization was analyzed by microscopy, round coverslips were placed into the wells prior to cell seeding. Subsequently, FITC-conjugated CIGB-552, was added to the cells to a final concentration of 5 μM or 50 μM and was incubated for 1 h at either 4 °C or 37 °C. For flow cytometry readout, culture medium was removed, cells were washed using phosphate-buffered saline (PBS) and surface-bound FITC-peptide was quenched with 0.4% (*w*/*v*) Trypan Blue solution. Cells were trypsinized and samples were acquired using Cyan ADP Flow Cytometer equipped with a 488 nm laser and a FL1 emission filter (530/40 nm). For data acquisition and analysis Summit v4.3 (Beckman Coulter, Fullerton, CA, USA) and FlowJo v.7.6.5 software (BD, Ashland, OR, USA) were used. For microscopy assays, cells were fixed in 4% (*w*/*v*) paraformaldehyde (PFA) for 15min, permeabilized using 0.2% (*v*/*v*) Tween 20 solution in PBS, and nuclei were stained using TOPRO-3 (1 μM, Life Technologies) and CellMask^TM^ membrane marker (1:2000, Life Technologies). 

### 4.5. Rab5A and Anti-COMMD1 Immunodetection

Cells were seeded in 12-well plates containing round coverslips (1×10^5^ cells/well) and cultured in either DMEM or RPMI supplemented with Glutamax, 10% (*v*/*v*) FBS, at 37 °C for 24 h. For early endosome staining CellLight^®^ Early Endosomes-RFP BacMam 2.0 (Life Technologies, Carlsbad, CA, USA) was used at a concentration of 20 particles per cell, and incubated with the cells overnight. Subsequently, FITC-CIGB-552 peptide was added to a final concentration of 50 μM and cells were incubated at 37 °C for 1h. Cells were fixed in 4% (*w*/*v*) PFA for 15min, permeabilized using 0.2% (*v*/*v*) Tween 20 solution in PBS and nuclei were stained with Hoescht 33342 (1:1000, Life Tech). For COMMD1 immunodetection, fixed cells were permeabilized and blocked with 2% (*w*/*v*) bovine serum albumin in PBS (BSA-PBS) for 1 h. Primary antibody, mouse monoclonal anti-COMMD1 (1:500, H00150684-M01, Abnova (Taipei, Taiwan) was diluted in BSA-PBS, added and incubated overnight at 4 °C. Three washing steps were performed using 0.2% (*v*/*v*) Tween 20 solution in PBS solution. Goat anti-Mouse IgG (H + L) secondary antibody, Alexa 488 conjugated (Life Technologies) diluted 1:1000 in BSA-PBS was added and incubated for 1:30 h. Finally, nuclei were stained with Hoescht 33342.

### 4.6. Protein Complementation Assay (PCA)

CIGB-552 is a synthetic peptide that possess modified amino acids (D amino acids) which cannot be translated inside cells. L2 peptide, which was chemically modified in its primary structure to generate the CIGB-552, was used for the protein complementation assay [5,6]. Cells were seeded in 12-well plates containing round coverslips (1 × 105 cells/well) and cultured in either DMEM or RPMI supplemented with Glutamax, 10% (*v*/*v*) FBS, at 37 °C for 24 h. Cells were co-transfected with the plasmids pCDNA3.1 Venus 1 and pCDNA3.1 Venus 2 each one containing either COMMD1 protein or L2 peptide. Transfection was performed with Lipofectamine 2000 (Thermo Fisher, Waltham, MA, USA) using a ratio of 1:2 DNA:lipofectamine (2 μg:1 μg). Transfection was carried out overnight, and cells were then fixed and stained using nuclear probe TOPRO-3 (Life Technologies) and fibrillar actin marker phalloidin Texas Red (Life Technologies).

### 4.7. Annexin V

Cells were seeded in 12-well plates (1 × 10^5^ cell/well), cultured for 24 h, collected and resuspended in Annexin V buffer (25mM HEPES, 140 mM NaCl, 1 mM EDTA and 2.5 mM CaCl_2_ pH 7.4. FITC-conjugated Annexin V (Life Technologies, Carlsbad, CA, USA) was added to the cells and incubated for 15 min. Samples were acquired in a Cyan ADP Analyzer (Beckman Coulter, Brea, CA, USA) equipped with 488 nm and 635 nm lasers. Fluorescence emissions were detected using band-pass filters 530/40. For each sample, 10,000 counts gated on a forward side scatter (FSC) versus side scatter (SSC) dot plot, excluding doublets were recorded. Summit v4.3 software was used for data acquisition and analysis.

### 4.8. Confocal Microscopy and Image Analysis

All images were taken using laser confocal microscope Leica TCS SP5 equipped with a 63X NA 1.42 oil immersion objective (Leica Microsystems, Wetzlar, Germany). In order to define internalization patterns, optical sections were scanned at intervals of 0.2–0.3 μm. Quantification of the number of fully loaded cells was performed using 10 single optical sections for each cell line by using the Manual Counting plugin provided in Icy 1.9.5.1v imaging software [29]. In order to assess fluorescence intensity both in cytoplasm and nuclei, images were taken in identical conditions (laser power, photomultiplier voltage, line and frame average and zoom) for all cell lines and 10 single optical sections were used for the analysis. Cytoplasm intensity values were obtained from applying 5 equal ROIs (region of interest) to each one of the images. Nuclei were segmented using the Active Contours plugin [30] provided by the Icy 1.9.5.1v imaging software. In both cases, data were collected by the imaging software and directly exported as an xls file.

### 4.9. Statistical Analysis

Data was expressed as the mean ± Standard Deviation of triplicates of one representative experiment, three independent experiments were executed. Statistic calculations were performed using Statistica software (STATSOFT, Hamburg, Germany). Differences were considered statistically significant when *p* < 0.05 using Mann-Whitney U Test.

## Figures and Tables

**Table 1:** CIGB-552 cytotoxicity in H460, HT-29 and MCF-7 cell lines (expressed by the IC_50_ value in μM); D-amino acids are indicated in small and italic characters.

Peptide	Sequence	H460	HT-29	MCF-7
CIGB-552	Ac-HARIK*p*TFRR*l*KWKYKGKFW	23 ± 8	166 ± 66	338 ± 39

**Table 2:** L2 and CIGB-552 peptide sequence.

Peptide	Sequence
L2	HARIKPTFRRLKWKYKGKFW
CIGB-552	Ac-HARIK*p*TFRR*l*KWKYKGKFW
